# On Vaccination

**Published:** 1880-12

**Authors:** 


### ON VACCINATION.

The Committee on Hygiene of the Medical Society of the County of Kings lay down the following propositions as a result of their investigations into the causes of alleged futile and bad results following vaccination:

1st. Vaccinate with only pure virus, animal or humanized, every child, when possible, before five months of age.

2d. The value of vaccination is lessened by lapse of time, so that re-vaccination is necessary between the tenth and fifteenth year.

3d. It is wisdom to vaccinate before an epidemic occurs, as has been repeatedly urged by ex-Sanitary Supt. Dr. Segur, before the public is excited, and when virus can be readily obtained. As the danger of small-pox is great, children who might not be considered in proper condition for vaccination at other times should be vaccinated during the presence of variola.

4th. Children should not be vaccinated during an eruption of teeth, the prevalence of an epidemic of diphtheria, in the hot weather, if it can be avoided, or where there is any skin eruption.

5th. The causes of "spurious vaccination in the Confederate army," as investigated by Prof. Joseph Jones, are interesting in this connection, viz:

1st. Lowered vitality—scorbutic condition.

2d. From abnormal lymph, from persons previously vaccinated or having eruptive diseases.

3d. Scabs or lymph undergoing decomposition, long carried about the person.

4th. Mingling vaccine virus with that of true variola, as in persons having varioloid.

5th. Virus from persons having erysipelas, pyæmia, gangrene and suppurating wounds.

6th. Lymph scabs, etc., from persons suffering from syphilis.

